# Pre-Compensation of Thermal Error for Laser-Assisted Diamond Turning

**DOI:** 10.3390/mi14101843

**Published:** 2023-09-27

**Authors:** Kaiyuan You, Guangyu Liu, Guangpeng Yan, Fengzhou Fang, Wei Wang, Li Du, Jiexiong Ding

**Affiliations:** 1 Yangtze Delta Region Institute (Quzhou), University of Electronic Science and Technology of China, Quzhou 324003, China; youkaiyuan@uestc.edu.cn (K.Y.); wangwhit@163.com (W.W.); 2 School of Mechanical and Electrical Engineering, University of Electronic Science and Technology of China, Chengdu 611731, China; lidu@uestc.edu.cn (L.D.); jxding@uestc.edu.cn (J.D.); 3 State Key Laboratory of Precision Measuring Technology and Instruments, Laboratory of Micro/Nano Manufacturing Technology (MNMT), Tianjin University, Tianjin 300072, China; liuguangyu@tju.edu.cn (G.L.); gpyan@tju.edu.cn (G.Y.)

**Keywords:** laser-assisted diamond turning, tool path, form accuracy, finite element analysis

## Abstract

The laser-assisted diamond turning (LADT) method can effectively improve the machinability of hard and brittle materials based on the laser heating effect, resulting in prolonged diamond tool life and better surface integrity. However, due to the incomplete absorption of laser beam energy within the workpiece cutting zone, simultaneous heating of the tool holder occurs, resulting in a structural thermal expansion that affects the workpiece form accuracy. In this article, the form accuracy of a LADT-machined workpiece was systematically studied. Accurate calculations of the tool shank and tool holder thermal fields and thermal expansion were performed using thermodynamic coupled finite element analysis. In addition, the LADT tool path was precisely pre-compensated by taking into account the structure expansion. The experimental results demonstrate that the form accuracy can be significantly improved with a pre-compensated tool path, which provides crucial technical support for achieving a high-precision finish on optical elements using the LADT method.

## 1. Introduction

Hard and brittle materials possess numerous advantages, including light weight, high strength, wear resistance, and high refractive index properties [1,2]. Consequently, typical tungsten carbide (WC), silicon (Si), and silicon carbide (SiC) hold immense application value in advanced optics and precision glass molding fields. Among them, binderless WC is a crucial mold material that is widely used in precision glass molding. High-performance glass lenses can be mass-produced in an efficient and economical manner using glass molding technology and high-quality optical molds made of binderless WC [3].

Traditional ultra-precision machining processes have significant restrictions and drawbacks [4,5]. It is challenging to maintain the machining efficiency, surface integrity, and residual stress state at the same time [6]. Ultra-precision grinding is currently the primary method used for machining binderless WC, which allows for optical surface machining with nanometric roughness and sub-micron form accuracy. However, the unsatisfactory subsurface integrity and surface tensile stress state limit the service life and performance of the mold insert. In order to reduce the influence of surface stress and the subsurface damage layer, it is essential to remove the machined affected layer through a subsequent polishing process, which significantly limits the machining efficiency and surface shape accuracy. At the same time, the feasibility of ultra-precision grinding is even compromised when the workpiece has a high-aspect-ratio micro-nano structure geometry [7]. The above problems can be solved by ultra-precision cutting methods, such as high-speed flying cutting, single point diamond turning, etc. These methods have unique advantages in terms of surface flexibility and machining efficiency, while also resulting in residual compressive stress on the workpiece surface. Furthermore, the depth of subsurface damage is greatly reduced compared with grinding, thereby helping to ensure device performance and service life. However, the machining of hard and brittle materials leads to severe diamond tool wear and surface cracking, which greatly limits the workpiece aperture size and surface quality [8]. Laser-assisted diamond turning (LADT) is one of the most effective machining methods for hard and brittle materials. It possesses widespread application prospects [9] and mainly utilizes the thermal softened effect to improve materials’ machinability. The feasibility of the LADT method has been verified in various hard and brittle materials, including silicon [10], zinc selenide [11], and tungsten carbide [12]. With the laser in-process heating assistance, diamond tool wear, finish quality, subsurface damage, and machining efficiency can be effectively improved compared with the conventional single-point diamond turning method [13]. However, it is challenging to ensure the sub-micron form accuracy of the workpiece using the LADT method. The incident laser beam will not only heat the cutting zone of the workpiece material, but also lead to an undesired temperature increase in the tool holder, which will seriously affect machining stability and tool path accuracy [14]; this has greatly limited the application of the LADT method.

Due to the increasing performance requirements of modern optical systems, higher requirements are put forward for the form accuracy of key components [15]. Optical elements with large form errors will lead to imaging distortion and restricted optical system performance [16]. The machined surface form accuracy is affected by several dominant factors, including tool path, tool edge waviness, lathe stability, etc. [17]. Among these factors, the tool path is the easiest to control and compensate [18]. The tool path accuracy will not only affect the form accuracy, but also affect the surface quality of complex optical elements [19]. Any deviation in the tool path will be directly transferred to the machined surface [20]. At present, published tool path research mainly focuses on traditional diamond turning. No LADT tool path investigation has been carried out. The system error introduced by the tool geometrical factors, including the nose radius and rake angle, can be compensated precisely [21,22,23]. However, there is no strategy to compensate the thermal drift of diamond tool in the LADT machining processing. It is urgent to propose a LADT tool path thermal compensation method.

The thermal deformation of optical elements is common for hot-forming technology, such as precision glass molding [24] and precision injection plastic molding [25]. In industrial mass production, the mold pre-compensation method is commonly used to eliminate the effect of material thermal deformation and ensure the form accuracy of the generated lens. The numerical analysis can significantly reduce the number of pre-compensation iterations and improve final lens form accuracy. Su et al. [26] established the finite element analysis (FEA)-assisted compensation procedure, which calculates the deformed lens’s profile precisely and obtains the pre-compensated mold insert surface form in advance. Zhang et al. [27] proposed an effective mold pre-compensation method based on mathematical analysis to eliminate the glass expansion and contraction influence in the precision glass molding process. In the same way, the structural thermal expansion effects of LADT machining can be eliminated by tool path pre-compensation. But to the best of the authors’ knowledge, no related study has been performed.

In this paper, we propose the LADT tool path pre-compensation method to improve the LADT-machined workpiece form accuracy. In Section 2, a systematic analysis of the effect of tool-setting errors on form accuracy is presented. The structural thermal expansion of tool holder and tool shank during the LADT machining process is precisely calculated using the FEA method, which enables thermal pre-compensation of the LADT tool path. Comparative experiments are described in Section 3. The experimental results in Section 4 successfully demonstrate that the proposed method can effectively improve the form accuracy of the LADT-machined workpiece.

## 2. Theoretical Approaches

### 2.1. Tool-Setting Error Influence

For accurate calculation of the tool path, it is essential to obtain the diamond tool position prior to the diamond turning process. However, tool-setting errors are inevitable due to the structural stress release drift and thermal expansion of the LADT machining. In this paper, we machined and analyzed an aspherical binderless WC surface with a diameter of 6 mm, which can be expressed as follows:

(1) $$z=\frac{cx^{2}}{1+\sqrt{1-(1+k)c^{2}x^{2}}}+a_{2}x^{2}+a_{4}x^{4}+a_{6}x^{6}+a_{8}x^{8}+a_{10}x^{10}+a_{12}x^{12}+a_{14}x^{14}$$

where *c* = 1/*R* is the radius of curvature of the aspherical vertex and *k* refers to the aspherical cone coefficient. The aspherical higher-order coefficients *a*_2_, *a*_4_, *a*_6_, *a*_8_, *a*_10_, *a*_12_, *a*_14_ are designed to correct the imaging aberration.

**Figure 1:**
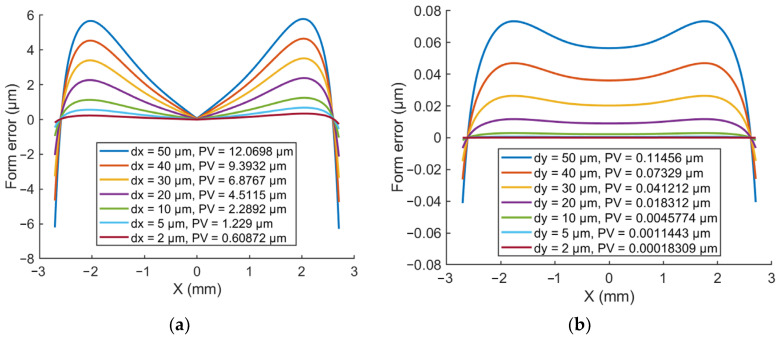
Effect of tool-setting errors in the (**a**) *X*-axis and (**b**) *Y*-axis directions on the aspherical workpiece form error.

In general, tool-setting errors in the *X*-axis and *Y*-axis directions directly affect the machined workpiece form accuracy. The tool-setting error in the *Z*-axis direction only affects the depth of a single cut for the conventional diamond turning. When the diamond tool possesses 50 μm, 40 μm, 30 μm, 20 μm, 10 μm, 5 μm, 2 μm setting errors in the *X*-axis and *Y*-axis directions, there will be a corresponding variable form error on the machined surface, as shown in Figure 1, although the theoretical calculation results show that *Y*-axis setting error has a much smaller effect on the surface form error compared with the *X*-axis error. It will leave a severe central cylindric or conical protrusion defect on the machined surface when the diamond tool is placed below or above the spindle center, respectively, which leads to a long iterative correction time in the subsequent polishing stage [28,29].

### 2.2. Structural Thermal Expansion

According to the laser path tracing simulation results [12], part of the laser beam will be reflected and irradiated on the tool shank. Thus, the thermal expansion of tool shank and holder is inevitable during the LADT process. To precisely obtain the thermal expansion trend during the LADT machining process, the three-dimensional thermodynamic coupled deformation is analyzed using FEA simulation. A tool shank made of WC and a tool holder made of stainless steel are assembled in the model, while thermal exchange, thermal radiation, and natural convective heat transfer are always taken into account. Considering the following experiment-adopted laser parameters, the local upper surface of the tool shank is heated by the equivalent 18 W laser simultaneously (at this time, the diamond tool emits 10 W). The initial temperature and ambient temperature are both set as 293 K. The simulation parameters are summarized in Table 1.

**Figure 2:**
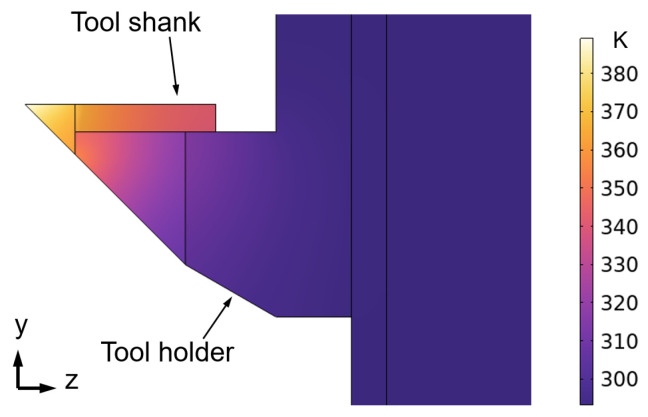
Thermal field of tool shank and tool holder during the LADT machining.

Since a 2 mm/min feedrate and a 10 mm aperture workpiece were used in the experiments, the total machining time of the workpiece was 150 s. There is always a dwell time of 30 s before the LADT machining process. In this way, the tool shank is heated for 180 s before the diamond tool reaches the workpiece center. Due to the thermal conductivity between contact surfaces, the tool holder temperature will also increase. As a result, the tool shank reaches a maximum temperature of 384 K near the diamond tool, as shown in Figure 2.

**Figure 3:**
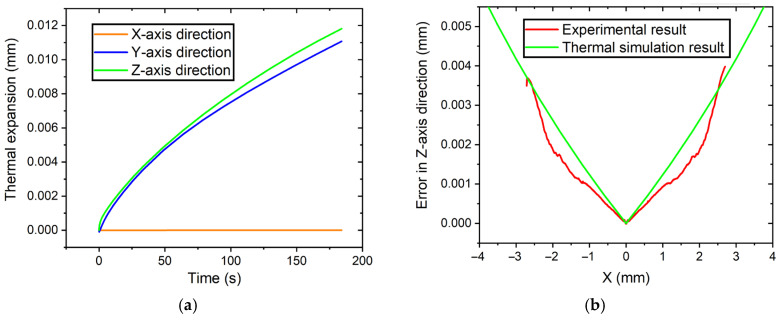
Simulation results: (**a**) diamond tool tip thermal drift, (**b**) the comparison between the thermal expansion in Z-direction and form error of LADT-machined workpiece.

The mechanical deformation during the 180 s laser heating was precisely calculated and analyzed. The simulation results indicate that the diamond tool will gradually expand in the *Y*-axis and *Z*-axis directions, which will severely affect the accuracy of the diamond tool set and machined surface form. Specifically, due to the symmetric design of the structure, there is no thermal drift along the *X*-axis direction for the diamond tool tip, and the diamond tool tip will expand 10.9 μm and 11.7 μm along the *Y*-axis direction and *Z*-axis direction, respectively, as shown in Figure 3a, which should be pre-adjusted in the tool fine setting process. Moreover, there is a great consistency between LADT-machined workpiece form error with uncompensated tool path and tool holder thermal expansion in the *Z*-axis direction, as shown in Figure 3b, which demonstrates the FEA simulation precision and also indicates that thermal expansion plays the dominant role in the LADT-machined surface form error. Furthermore, the residual difference in Figure 3b is mainly attributed to the tool-setting error, measurement error of the tool nose radius, and tool edge waviness.

## 3. Experimental Setup

**Figure 4:**
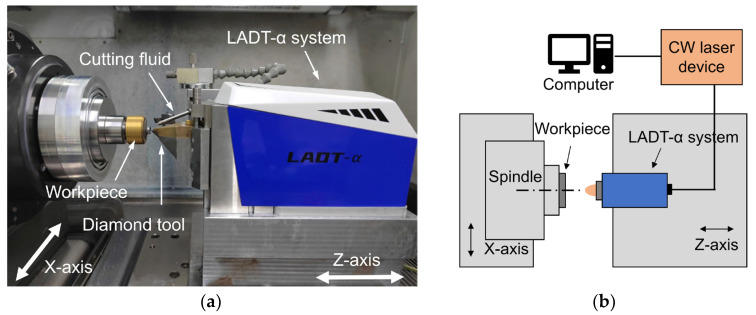
LADT (**a**) experimental setup and (**b**) schematical diagram.

The comparative LADT experiment was conducted based on the ultra-precision 3-axis lathe and self-developed LADT-α system, as shown in Figure 4. The LADT-α system can generate a 1064 nm wavelength continuous-wave (CW) laser beam. After optical collimating and focusing, a 170 μm diameter laser beam can be guided to the workpiece cutting area through a transparent diamond tool.

A binderless WC workpiece with a 10 mm overall aperture and 6 mm aspherical aperture was machined by a brand-new cylindrical diamond tool with a 0.3 mm nose radius and −35° rake angle. The edge-to-center diamond tool feed direction with a feed rate of 2 mm/min and a rotation speed of 2000 rpm was used in the experiments. According to the numerical simulation results, the vertical expansion height of 10.9 μm was pre-adjusted before the final turning. Cutting fluid was used to promote lubrication and reduce diamond tool wear. For clarity, the experimental parameters are summarized in Table 2.

**Figure 5:**
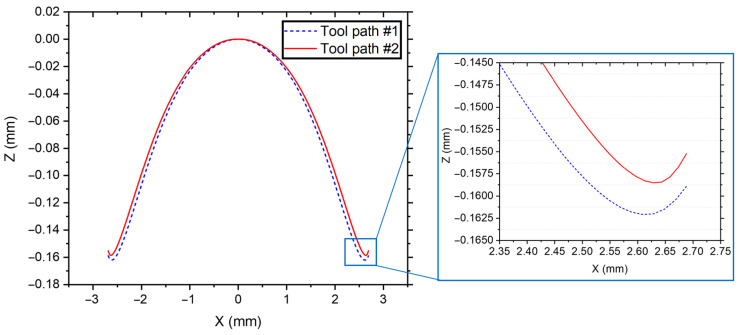
Tool path comparison between original tool path #1 and thermal pre-compensated tool path #2.

In the LADT experiments, tool paths were generated in the following two steps. Firstly, the diamond tool geometries, including nose radius and negative rake angle, were all compensated based on the machined aspherical surface form, which can directly generate tool path #1. Secondly, the simultaneous structural thermal expansion along the *Z*-axis direction can be determined from the results of FEA simulation and the experimental feed rate. In this way, tool path #2 can be obtained by subtracting the simultaneous Z-expansion values from tool path #1, as shown in Figure 5.

## 4. Results and Discussion

**Figure 6:**
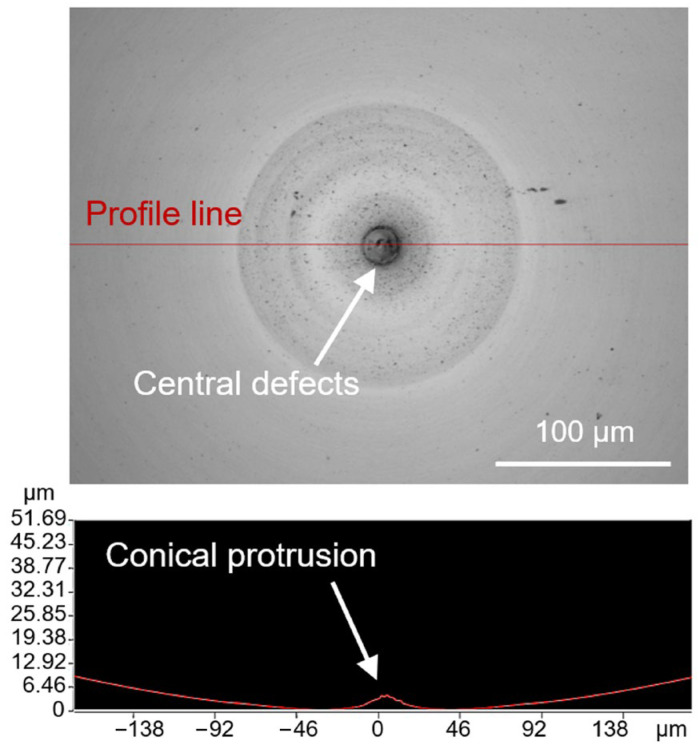
Workpiece central protrusion defects caused by diamond tool tip thermal drift.

Due to the continuous heating of the tool shank by the reflected laser beam during the LADT machining process, the tool shank and tool holder will expand in the *Y*-axis direction, which directly affects the accuracy of the diamond tool set position. If the thermal expansion has not been compensated in advance, the diamond tool will always be higher than the workpiece center, leaving a conical protuberance defect in the central region, as shown in Figure 6. Specifically, the deteriorated finish quality in the region around the central protrusion can be attributed to the unstable cutting state when the diamond tool flank face squeezes against the central material.

**Figure 7:**
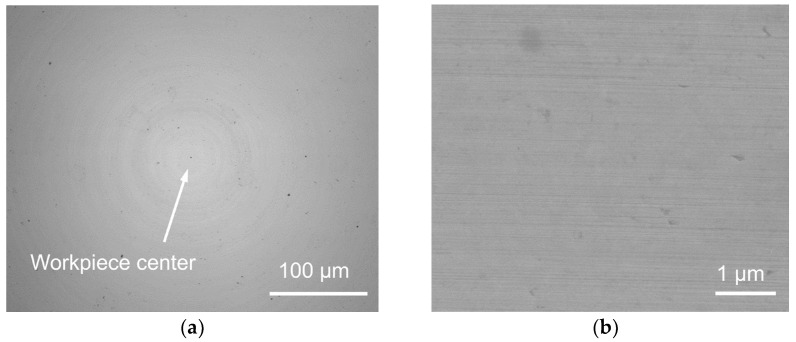
LADT-machined binderless WC mold insert surface morphology with a pre-compensated diamond tool height in the (**a**) central region, and (**b**) fringe region.

Two binderless WC mold inserts were machined using the LADT method with raw tool path #1 and pre-compensated tool path #2, respectively. The diamond tool tip height was pre-adjusted to 10.9 μm to compensate the vertical thermal expansion before both turning passes. Thus, only a slight central defect can be observed on the machined workpiece, as shown in Figure 7a, indicating the tool-setting error in the *Y*-axis direction has been basically eliminated. Furthermore, a stable machining state can be maintained during the LADT process, thereby resulting in a homogeneous surface, as shown in Figure 7b.

**Figure 8:**
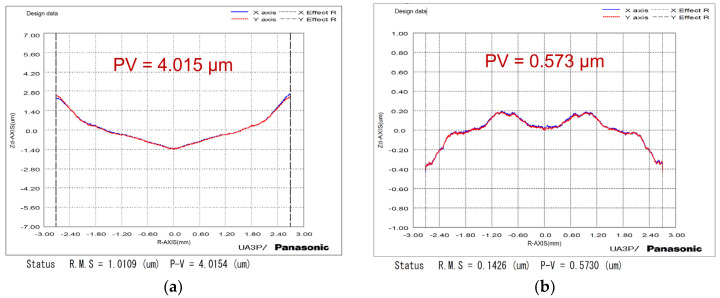
Workpiece form errors machined by LADT with (**a**) raw tool path #1, and (**b**) pre-compensated tool path #2.

The machined workpieces were estimated by a form measurement instrument (UA3P-300). The diamond probe with a radius of 2 μm was used to scan the optics at a scanning speed of 0.2 mm/s. There is a large difference in form accuracy between the tool path #1 and tool path #2 machined surfaces. The large form error PV value of 4.015 μm is unqualified, as shown in Figure 8a, using tool path #1, and there is a great consistency between the form error and the diamond tool thermal drift in the *Z*-axis direction, as shown in Figure 3b. Because thermal deformation was pre-compensated into toolpath #2, the workpiece form accuracy can be effectively improved to a PV value of 0.573 μm, as shown in Figure 8b. Moreover, there’s no obvious profile fluctuation in the central area, demonstrating that the diamond tool tip coincides with the workpiece center by pre-adjusting the expansion height, which is consistent with Figure 7a.

Comparative experimental results demonstrate the feasibility of the proposed LADT tool path pre-compensation method, although the form error of the aspherical surface can be improved by tool path compensation based on the machined surface profile measurement results. The pre-compensated tool path generation method can effectively save at least 1 turning pass, which is of interest for diamond tool life in the field of hard and brittle material machining. In addition, the proposed LADT pre-compensated tool path generation method provides a significant technique for freeform surface machining, which requires first-turn pass completion accuracy.

## 5. Conclusions

In this study, the LADT tool path pre-compensation method has been developed to eliminate the effect of thermal deformation on the form accuracy of machined surfaces. A systematic analysis and comparison of the effect of the tip drift of the diamond tool on the form accuracy was performed. The tool path is precisely pre-compensated based on the results of FEA thermal expansion calculation. Ideal experimental results successfully demonstrate the feasibility of the proposed method. The main conclusions can be summarized as follows:

1. The tool shank and tool holder will be heated to 384 K, resulting in large thermal drifts in the diamond tool tip of 10.9 and 11.7 μm in the *Y*-axis and *Z*-axis directions, respectively.
2. The workpiece central conical protrusion defects can be effectively eliminated by tool-setting height pre-compensation according to the FEA simulation results.
3. The form accuracy of the machined aspherical workpiece is effectively improved by 85.7% to a PV of 0.573 μm using the pre-compensated tool path #2.

## Figures and Tables

**Table 1:** The FEA simulation parameters.

Parameters	Value
Tool shank material	WC
Tool shank thermal expansivity	4.5 × 10^−6^ 1/K
Tool holder material	Stainless steel
Tool holder thermal expansivity	12.6 × 10^−6^ 1/K
Initial temperature	293 K
Laser power	18 W
Laser beam diameter	170 μm
Emissivity	0.8
Convection coefficient	10 W/(m^2^·K)

**Table 2:** The LADT experimental parameters.

Parameters	Value
Workpiece material	Binderless WC
Workpiece aperture	10 mm
Surface form	Aspherical (Convex with edge inversion)
Tool nose radius	0.3 mm
Tool rake angle	−35°
Feedrate	2 mm/min
Laser power	10 W
Cutting direction	Edge-to-center

## Data Availability

The data presented in this study may be available from the corresponding author upon reasonable request.
